# Do Conduct Problem Pathways Differ for Black and Minority Ethnic Children in the UK? An Examination of Trajectories from Early Childhood to Adolescence

**DOI:** 10.1007/s10964-019-01116-w

**Published:** 2019-09-03

**Authors:** Leslie Morrison Gutman

**Affiliations:** University College London, 1-19 Torrington Place, London, WC1E 7HB United Kingdom

**Keywords:** Conduct problems, Group-based trajectories, Black and minority ethnic, Britain, Childhood, Adolescence

## Abstract

A substantial body of evidence has examined developmental pathways into and out of conduct problems. However, there is a dearth of research examining whether the same conduct problem pathways are evident in minority ethnic, as in white, populations. Drawing on the UK Millennium Cohort Study (MCS), a nationally representative longitudinal study of children born between 2000 and 2002, this study examines differences in group-based trajectories of conduct problems according to broad categories of ethnicity. Using pathways identified in a prior study (*n* = 17,206, 49% female, 18% ethnic minority), including persistently high (8%), childhood-limited (23%), adolescent-onset (13%), and low (56%), significant ethnic differences were found. As a result, trajectories of conduct problems were identified separately for Asian, black, mixed ethnicity, and white children. For Asian, black, and mixed ethnicity children, three trajectories were identified: persistently high, childhood-limited, and low, but not adolescent-onset. Although these pathways have similar labels, their patterns and shapes seem to differ among the three ethnic groups. For white children, the same four trajectory groups were identified as in the prior study. Risk factors also differed among the groups according to ethnicity, although a worse child-parent relationship was a significant predictor of the higher problem trajectories for all ethnic groups. Overall, the findings suggest that black and minority ethnic children may follow different developmental pathways of conduct problems than white children, particularly during adolescence, having implications for service use and early intervention.

## Introduction

Conduct problems refer to behaviours under the conduct-oppositional spectrum, including those that are defiant, antisocial, and/or potentially harmful to others such as lying, stealing, physical aggression, and rule-breaking (American Psychiatric Association 2013). Research shows that conduct problems persisting through childhood and adolescence are a precursor to a wide range of adverse outcomes, not only in childhood, but throughout the life course and even extending into succeeding generations (see Gutman et al. 2018a, for a review). A substantial body of evidence has examined developmental pathways of conduct problems (see Bevilacqua et al. 2017, for a review). However, there is a dearth of research examining whether the same heterogeneous conduct problem pathways are evident in black and minority ethnic, as in white, children. Such an investigation, using population-based samples representing a wide socio-economic stratum, is essential in tailoring evidence-based and culturally-appropriate interventions. Using group-based trajectories of conduct problems from ages 3 to 14 years previously identified in the UK Millennium Cohort Study (MCS) (Gutman et al. 2019), this study aims to fill this gap by examining ethnic differences in these heterogeneous trajectories and their early risk factors.

### Developmental Trajectories of Conduct Problems

Developmental taxonomy theory has enlightened our understanding of the mechanisms underlying the emergence and continuity of conduct problems in childhood and adolescence. Moffitt (1993) identified two pathways of conduct problems based on age of onset and developmental course; differentiating between life-course, persistent and adolescent-limited conduct problems. Those following a life-course, persistent pathway (5 to 10%) show persistent and high levels of conduct problems from early childhood (i.e., before age 10) through childhood, exhibiting antisocial behaviour through adolescence and into adulthood (Broidy et al. 2003).

Adolescent conduct problems (15 to 30%), on the other hand, appear primarily from ages 10 to 17 (Moffitt 2006). Those on this trajectory are considered to be rebellious teenagers, who mature out of their antisocial behaviour in preparation for adulthood; this pathway has thus been labelled “adolescent-limited” (Moffitt 2006; Odgers et al. 2008). However, research has shown that adolescent conduct problems are not necessarily restricted to adolescence (Kretschmer et al. 2014; Sentse et al. 2017). Therefore, the term “adolescent-limited” may be misleading and rather should be referred to as “adolescent-onset” (Gutman et al. 2019; Sentse et al. 2017).

In addition to the life-course and adolescent-limited trajectories, advances in statistical modelling has enabled the identification of another pathway: childhood-limited or early-onset, desisting conduct problems. This group begins with high levels of conduct problems, which decrease in later childhood, often resembling the low group by adolescence (Odgers et al. 2008). Numerous studies utilising different age ranges, populations, and measures have supported the identification of these four conduct problem trajectories: persistent, adolescent-onset, childhood-limited, and low (see Bevilacqua et al. 2017, for a review).

### Ethnic Differences in Conduct Problem Trajectories

Studies in both the US and UK have shown ethnic variation in the prevalence of conduct problems (Cavendish et al. 2012; Gutman et al. 2015; Miner and Clarke-Stewart 2008). In the US, research suggests that African American, Asian American, and Hispanic youth are more likely to be diagnosed by clinicians with disruptive behaviour disorder and conduct-related problems than non-Hispanic, white youth (Mak and Rosenblatt 2002; Nguyen et al. 2007). In Britain, however, evidence indicates that most black and minority ethnic children and adolescents enjoy better or similar mental health overall compared to white children and adolescents (Goodman et al. 2008; Green et al. 2005), which is not explained by socio-economic circumstances, family type, social support, or perceived parenting (Klineberg et al. 2006; Maynard and Harding 2010; Maynard et al. 2007). Among black and minority ethnic groups, a systematic review of UK studies showed Indian, black African and Pakistani children as having an advantage in terms of fewer behavioural disorders, while mixed white/Black Caribbean children show relatively more behavioural problems in comparison to white children (Goodman et al. 2008).

There may also be age-related trends. Studies in the UK have found that black and minority ethnic adolescents generally show fewer mental health problems than white adolescents (Astell-Burt et al. 2012; Fagg et al. 2006; Goodman et al. 2010). Evidence from the Millennium Cohort Study indicates that Indian, Pakistani/Bangladeshi, and black children appear to have fewer conduct problems at age 11 than white and mixed ethnicity children (Gutman et al. 2015). Another study using the Millennium Cohort Study indicates that children of mixed ethnicity have lower rates of problematic behaviours than their non-mixed counterparts at age 3 years, but experience a greater rate of increase during early adolescence, from ages 7 to 11 years (Zilanawala et al. 2018). According to the authors, these shifts may be explained by potential conflicts between personal and social identities that emerge during the adolescent period for children of mixed identity.

When examining group-based trajectories of conduct problems, some US studies have identified heterogeneous pathways using ethnically diverse samples (e.g., Alink and Egeland 2013; Shaw et al. 2012; Xie et al. 2011); however, these have not assessed ethnic group differences. In the UK, one study used the Millennium Cohort Study to analyse ethnic variation in the prevalence of conduct problem trajectories from 3 to 14 years, finding that black and minority ethnic children were less prevalent in the persistently high and adolescent-onset conduct problem groups than white children (Gutman et al. 2019). However, no studies to date have examined ethnic differences in children’s conduct problem trajectories in terms of their number, shape, and rate of change. This is a much-needed step, as we do not know whether the developmental taxonomy applies equally among black and minority ethnic as white children. Given the ethnic diversity of Britain with 21% ethnic minorities under age 18 (Cabinet Office 2018), the identification of heterogeneous pathways of conduct problems for black and minority ethnic children may further inform the understanding of ethnic differences in etiology, health inequalities, service use, and whom and when to target for early intervention.

### Early Risks of Conduct Problem Trajectories

Risk factors have also been shown to differentiate pathways of conduct problems, each involving distinct combinations of factors (Frick 2012). This study focuses on the earliest precursors that may distinguish children following high conduct problem trajectories from those on a low problem pathway. Research suggests that early socio-economic disadvantage is a risk factor for high conduct problem trajectories, no matter the age of onset (Barker and Maughan 2009; Gutman et al. 2019). In addition, those on high conduct problem trajectories often live in households characterised by family instability, financial hardship, and harsh, negative parenting in comparison to the low conduct problem group (Frick and Viding 2009; Shaw 2013). The high conduct problem pathways have also been associated with a greater likelihood of being born to a teenage mother and growing up in a single-parent family (Barker and Maughan 2009; Gutman et al. 2018b, 2019). The persistently high group, in particular, has been found to have the highest levels of risk in early childhood, particularly socio-economic disadvantage, while the childhood-limited and adolescent-onset groups show intermediate levels of risk, with earlier risks more likely for the childhood-limited group and later risks emerging for the adolescent-onset group (Gutman et al. 2019). Although evidence indicates that some black and minority ethnic children are more likely to live in families facing social inequalities such as low income (Department for Work and Pensions 2018) and maternal mental illness (National Health Service 2018) compared to their white counterparts, there exists almost no research, examining whether these factors are significant predictors of heterogeneous conduct problem trajectories among black and minority ethnic children living in the UK.

## Current Study

Using the Millennium Cohort Study, a nationally representative sample of children born around the new millennium in the UK, four trajectories of conduct problems from ages 3 to 14 years (persistently high, childhood-limited, adolescent-onset, and low) were identified in a prior study (Gutman et al. 2019). This study aims to (a) assess ethnic differences in these pathways, (b) identify ethnic-specific trajectories of conduct problems, and (c) examine early predictors of these ethnic-specific trajectories. Using broad categories of ethnicity based on the UK 2011 Census (Office for National Statistics 2009), this study examines whether there are statistically significant differences among the initial values (i.e., intercepts) and rates of change (i.e., slopes) of these pathways between Asian and white children, black Caribbean/African and white children, and mixed ethnicity and white children. If statistically significant differences emerge, then ethnic-specific trajectories are identified. Further examined are early markers; those factors shown to differentiate pathways of conduct problems in prior studies including socio-economic disadvantage; maternal factors such as teen pregnancy and depressive symptoms; single-parent family; and the child-parent relationship (Barker and Maughan 2009; Gutman et al. 2019; Frick and Viding 2009; Shaw 2013).

Given the dearth of research examining ethnic differences among conduct problem trajectories and their early predictors, no firm hypotheses are offered. However, it is possible that some pathways may be identified for one ethnic group, but not for the other. Despite the importance of estimating ethnic-specific trajectories if statistically significant differences are found, it is possible to identify groups which are not clinically meaningful. For example, a high problem trajectory group of black and minority ethnic children may be identified that has relatively lower levels of conduct problems compared to a high problem group of white children. To remedy this, clinically meaningful age cut-offs based on national norms from England are used, which have been shown to predict strongly later conduct disorder diagnoses (Goodman et al. 2000).

## Method

### Study Sample

MCS is a nationwide multi-purpose longitudinal study following children born in all four countries of the UK between September 2000 and January 2002 (Joshi and Fitzsimons 2016). The survey has a complex clustered and disproportionately stratified design. The clusters were electoral wards, and the strata oversampled the three smaller countries of the UK (to allow for analysis within them), wards with high child poverty in all four countries, and wards with high minority ethnic populations in England. Within a ward, all children born in the target period were eligible, regardless of birth order. Data are so far available from six sweeps of interviews with the families. The first survey, MCS1 (child age 9 months) was in the field mainly in 2001, MCS2 (age 3 years) mainly during 2004, MCS3 (age 5 years) mainly during 2006, and MCS4 (age 7 years) mainly during 2008. MCS5 (age 11 years) collected data mainly in 2012 when the cohort children were in their last year of primary school. MCS6 (age 14 years) collected data mainly in 2015 when they were in secondary school. The main informants were overwhelmingly the natural mothers (99% at MCS1, 96% by MCS5).

The number of families who have been interviewed at least once is 19,244, including 692 families in England who were not recruited until MCS2. If these cases are counted, the initial response rate was 71%. In this study, the sample included one child per family, excluding children who were the second or third in sets of twins and triplets. This study included 17,206 children with data on parent-reported conduct problems in two or more surveys from MCS2 to MCS6. For the most part, the 2,037 children excluded from this study due to missing data belonged to families who were non-responsive after MCS1. These non-responsive families were more likely to be teenage and single parents, have lower household income and education, and live in social housing compared to the responsive families. Black and ethnic minorities were more likely to be missing than white respondents. The MCS survey team has developed attrition weights to correct for biases due to non-response, alongside the sample weights which take into account the complex sample design (Hansen 2014, 18–22).

Ethnic categories were constructed using mother’s reports of the child’s ethnicity at MCS1 and MCS2 and were based on the UK 2011 Census categories (Office of National Statistics 2009), including 82% white; 10.5% Asian (2.5% Indian, 5% Pakistani, 2% Bangladeshi, 1% other Asian), 4% black (1.5% black Caribbean, 2% black African, 1% other black), 3% mixed ethnicity, and 0.5% other ethnicities.

### Measures

#### Conduct problems

This was measured using the Strengths and Difficulties Questionnaire (SDQ) (Goodman 1997, 2001), completed by the mother when the child was 3, 5, 7, 11, and 14 years-old. In the MCS, construct, convergent, discriminant, and predictive validity have been established for age 3, 5, and 7 SDQ subscales, showing good internal reliability with alphas ranging from 0.77 to 0.82 for conduct problems (Croft et al. 2015). At ages 11 and 14 years, the internal reliabilities of conduct problems are acceptable, with alphas of 0.62 and 0.64, respectively. The questionnaire assesses conduct problems in the past 6 months using five items: (1) often has temper tantrums or hot tempers; (2) generally obedient, usually does what adults request; (3) often fights with other children or bullies them; (4) often lies or cheats; and (5) steals from home, school or elsewhere. To ensure that they are clinically meaningful, continuous scores were converted using SDQ bandings based on externally given UK norms calculated from a survey population comprised of children and adolescents aged 5 to 15 (Meltzer et al. 2009), where 10% in that reference sample with the highest scores were considered to be at high risk of conduct problems (0 = not high risk; 1 = high risk).

#### Early predictors

These were measured when the child was 9 months-old, with the exception of Maternal Depressive Symptoms and Child-Parent Relationship Scale, which was measured when the child was 3 years-old.

##### Single parent families

Families headed by a single parent (1 = single-parent families; 0 = two parents).

##### Highest household education

The highest educational level of either the mother or father, where applicable (0 = no formal qualification; 5 = professional degree).

##### Family income

A measure of net family income was derived for general use by the survey team (Ketende and Joshi 2008). This had imputed missing values, adjusted for family size (equivalized), and for survey weighting, dividing families into five equally sized income bands (1 = lowest quintile; 5 = highest quintile).

##### Social housing

Families currently renting from local authorities or housing associations (1 = living in social housing; 0 = not living in social housing).

##### Maternal depressive symptoms (alpha = 0.88)

A mean score of mother’s responses to 6 items from the Kessler Scale (Kessler et al. 2002) was calculated. Items include, “During the last 30 days, about how often did you feel hopeless?” and “During the last 30 days, about how often did you feel that everything was an effort?” (1 = none of the time; 5 = all of the time).

##### Child-parent relationship scale (CPRS)

*Short Form* (*alpha* = 0.90) was used to calculate a mean score of mother’s responses to 15 items about the child-parent relationship (Pianta 1995). The CPRS includes statements, such as: “My child will seek comfort with me” and “My child and I always seem to be struggling with each other” (reversed) (1 = definitely does not apply; 5 = definitely applies). The items measure the mother’s positive feelings and beliefs about her relationship with her child and about the child’s behaviour toward her.

### Statistical Analyses

Group-based trajectory analysis in STATA TRAJ (Jones and Nagin 2013) was used to model trajectories as a function of each child’s age measured in months at each interview. Group-based trajectory modelling is a specialised form of finite mixture modelling (see Nagin 2005; Nagin and Odgers 2010). Full Information Maximum Likelihood (FIML) estimated the model parameters, thereby including every case with at least two maternal ratings (Schafer and Graham 2002). Binary logit distribution was specified as conduct problems is a dichotomous outcome. To establish the best fitting solution, a range of fit indicators were examined including the lowest absolute Bayesian Information Criterion (BIC), the average posterior probability of group membership (0.70 being acceptable), a close correspondence between the estimated probability of group membership, and the proportion assigned to that group based on the posterior probability of group membership (Nagin 2005).

Using the four-group, cubic model previously identified, ethnic differences in the intercept and slopes of the low (56%), persistently high (8%), childhood-limited (23%), and adolescent-onset (13%) trajectories were examined. Separate models were run to test for ethnic differences between Asian and white children, black and white children, and mixed ethnicity and white children using ethnicity (intercept), time-varying ethnicity by age (linear slope), and time-varying ethnicity by age-squared (quadratic) as covariates (Jones and Nagin 2013). The other ethnicity group was not tested due to a small n. Group differences in the cubic slope were also not tested due to limited degrees of freedom. In the case of significant ethnic differences, separate models for each ethnic group were run.

In order to account for the complex clustered and stratified survey design of MCS, *svy* in STATA was used in the following stages of the analyses. First, the proportions and standard deviations of the early predictors and conduct problems by ethnic group were assessed (see Table 1). Second, using subgroup analysis, the proportions and standard deviations of the early predictors by trajectory group were examined (see Tables 2–5). For both of these steps, univariate regressions were run followed by post-hoc tests to compare all possible pairwise differences among the groups using the Bonferroni correction.

**Table 1:** Ethnic differences among conduct problems and early predictors

	Asian Children	Black Children	Mixed Children	White Children	*F-*test
	Mean (SD)	Mean (SD)	Mean (SD)	Mean (SD)	
Measures					
Conduct problems-age 3	0.22 (0.53)	0.16 (0.43)	0.20 (0.39)	0.18 (0.38)	*F* (4, 14691) = 2.23
Conduct problems-age 5	0.11 (0.41)	0.08 (0.34)	0.10 (0.30)	0.09 (0.28)	*F* (4, 14654) = 1.16
Conduct problems-age 7	0.09 (0.37)	0.09 (0.35)	0.11 (0.31)	0.09 (0.28)	*F* (4, 13384) = 1.36
Conduct problems-age 11	0.06 (0.31)^a^	0.07 (0.30)^ab^	0.09 (0.29)^ab^	0.10 (0.29)^b^	*F* (4, 12720) = 3.94**
Conduct problems-age 14	0.09 (0.37)	0.09 (0.34)	0.11 (0.30)	0.10 (0.29)	*F* (4, 11260) = 0.27
Early predictors					
Household highest education	2.85 (1.89)^a^	3.22 (1.59)^b^	3.23 (1.21)^b^	3.16 (1.10)^b^	*F* (4, 14762) = 8.50***
Family income	2.26 (1.62)^a^	2.41 (1.72)^a^	2.87 (1.53)^b^	3.17 (1.34)^c^	*F* (4, 16446) = 123.06***
Social housing	0.17 (0.49)^a^	0.62 (0.58)^b^	0.36 (0.47)^c^	0.20 (0.39)^a^	*F* (4, 16461) = 77.50***
Single-parent household	0.07 (0.32)^a^	0.41 (0.59)^b^	0.26 (0.43)^c^	0.12 (0.32)^d^	*F* (4, 16485) = 60.64***
Teenage mother	0.04 (0.24)^a^	0.05 (0.25)^ab^	0.08 (0.26)^b^	0.07 (0.25)^b^	*F* (4, 17096) = 11.77***
Maternal depressive symptoms	4.19 (5.46)^a^	3.68 (4.98)^ab^	3.48 (3.75)^ab^	3.08 (3.45)^b^	*F* (4, 13503) = 10.76***
Child-parent relationship	4.32 (0.64)^a^	4.49 (0.50)^b^	4.43 (0.46)^b^	4.42 (0.45)^b^	*F* (4, 13719) = 7.63***

Post-hoc analyses using Bonferroni’s method identified significant pairwise comparisons (*p* < 0.05) between groups, shown when group means do not share any similar superscripts

*p < 0.05; **p < 0.01; ***p < 0.001

**Table 2:** Mean differences in gender and early predictors by trajectory group for Asian children

	Trajectory Group						*F-*test
Variables	Low		Childhood-Limited		Persistent		
	Mean	SD	Mean	SD	Mean	SD	
Male	0.49	0.50	0.54	0.50	0.57	0.52	*F* (2, 1618) = 1.65
Household highest education	2.94^a^	1.48	2.61^ab^	1.48	2.43^b^	1.45	*F* (2, 1228) = 5.22**
Family income	2.32^a^	1.28	2.14^ab^	1.21	1.84^b^	1.00	*F* (2, 1601) = 10.50***
Social housing	0.16	0.37	0.22	0.42	0.18	0.40	*F* (2, 1607) = 1.52
Single-parent	0.06	0.24	0.07	0.25	0.12	0.34	*F* (2, 1618) = 1.77
Teenage mother	0.03	0.18	0.05	0.22	0.05	0.24	*F* (2, 1729) = 1.23
Maternal depressive symptoms	3.73^a^	4.04	5.37^b^	4.80	6.65^b^	5.08	*F* (2, 762) = 14.07***
Child-parent relationship	4.43^a^	0.44	4.03^b^	0.47	3.82^b^	0.57	*F* (2, 774) = 44.53***

Post-hoc analyses using Bonferroni’s method identified significant pairwise comparisons (*p* < 0.05) between groups, shown when group means do not share any similar superscripts

*p < 0.05; **p < 0.01; ***p < 0.001

**Table 3:** Mean differences in gender and early predictors by trajectory group for black children

	Trajectory Group						*F-*test
Variables	Low		Childhood-Limited		Persistent		
	Mean	SD	Mean	SD	Mean	SD	
Male	0.48^a^	0.50	0.66^b^	0.47	0.79^b^	0.40	*F* (2, 564) = 7.76***
Household highest education	3.30	1.62	2.89	1.44	2.89	1.40	*F* (2, 433) = 1.74
Family income	2.49	1.44	2.14	1.44	2.03	1.36	*F* (2, 562) = 1.95
Social housing	0.60	0.49	0.73	0.44	0.69	0.45	*F* (2, 562) = 1.94
Single-parent	0.39	0.49	0.48	0.50	0.58	0.48	*F* (2, 564) = 2.47
Teenage mother	0.04	0.20	0.05	0.22	0.07	0.24	*F* (2, 617) = 0.21
Maternal depressive symptoms	3.09^a^	3.65	5.64^b^	5.24	5.51^b^	5.07	*F* (2, 334) = 4.90**
Child-parent relationship	4.59^a^	0.67	4.23^b^	0.42	4.04^b^	0.52	*F* (2, 344) = 19.51***

Post-hoc analyses using Bonferroni’s method identified significant pairwise comparisons (*p* < 0.05) between groups, shown when group means do not share any similar superscripts

*p < 0.05; **p < 0.01; ***p < 0.001

**Table 4:** Mean differences in gender and early predictors by trajectory group for mixed ethnicity children

	Trajectory Group						*F-*test
Variables	Low		Childhood-Limited		Persistent		
	Mean	SD	Mean	SD	Mean	SD	
Male	0.45^a^	0.50	0.74^b^	0.44	0.57^ab^	0.50	*F* (2, 478) = 7.81***
Household highest education	3.15^a^	1.62	2.65^ab^	1.43	2.21^b^	1.07	*F* (2, 420) = 8.88***
Family income	2.62^a^	1.52	2.13^ab^	1.37	1.70^b^	1.05	*F* (2, 477) = 8.29***
Social housing	0.39^a^	0.49	0.54^ab^	0.50	0.76^b^	0.43	*F* (2, 477) = 10.92***
Single-parent	0.28^a^	0.45	0.53^b^	0.50	0.51^b^	0.51	*F* (2, 478) = 9.57***
Teenage mother	0.08	0.28	0.15	0.36	0.18	0.38	*F* (2, 514) = 2.71
Maternal depressive symptoms	3.63	3.87	5.03	4.75	4.12	5.70	*F* (2, 375) = 1.95
Child-parent relationship	4.47^a^	0.44	4.08^b^	0.44	4.03^b^	0.58	*F* (2, 344) = 22.33***

Post-hoc analyses using Bonferroni’s method identified significant pairwise comparisons (*p* < 0.05) between groups, shown when group means do not share any similar superscripts

*p < 0.05; **p < 0.01; ***p < 0.001

**Table 5:** Mean differences in gender and early predictors by trajectory group for white children

	Trajectory Group								*F-*test
Variables	Low		Childhood-Limited		Adolescent-onset		Persistent		
	Mean	SD	Mean	SD	Mean	SD	Mean	SD	
Male	0.49^a^	0.49	0.54^b^	0.51	0.56^bc^	.50	0.61^c^	0.51	*F* (3, 13726) = 17.22***
Household highest education	3.27^a^	1.09	2.93^b^	1.17	2.87^b^	1.15	2.55^c^	1.19	*F* (3, 12596) = 99.56***
Family income	3.37^a^	1.32	2.79^b^	1.42	2.75^b^	1.41	2.21^c^	1.31	*F* (3, 13706) = 223.82***
Social housing	0.15^a^	0.36	0.29^b^	0.47	0.38^b^	0.48	0.46^c^	0.52	*F* (3, 13713) = 138.98***
Single-parent	0.10^a^	0.29	0.17^b^	0.39	0.16^b^	0.37	0.28^c^	0.47	*F* (3, 13726) = 55.70***
Teenage mother	0.05^a^	0.22	0.10^b^	0.30	0.10^b^	0.31	0.16^c^	0.38	*F* (3, 14121) = 35.05***
Maternal depressive symptoms	2.54^a^	2.91	4.33^b^	4.32	4.03^b^	4.07	5.55^c^	5.37	*F* (3,11977) = 148.23***
Child-parent relationship	4.55^a^	0.38	4.08^b^	0.36	4.28^c^	0.48	3.90^d^	0.50	*F* (3, 12161) = 695.77***

Post-hoc analyses using Bonferroni’s method identified significant pairwise comparisons (*p* < 0.05) between groups, shown when group means do not share any similar superscripts

*p < 0.05; **p < 0.01; ***p < 0.001

## Results

### Ethnic Differences in Conduct Problems and Early Predictors

Table 1 presents the means and standard deviations of all variables shown according to the child’s ethnicity. The results show that white children had a higher probability of clinically meaningful conduct problems at age 11 compared to Asian children. Asian children had lower household education than black, mixed ethnic, and white children; Asian, black, and mixed ethnic children had lower family income than white children. Black children had the highest rate of living in social housing and with single-parent families compared to the other groups. Mothers of Asian children reported more depressive symptoms than white mothers and the least positive child-parent relationship of all the groups.

### Trajectories of Conduct Problems

Significant differences were found between Asian and white children and between black and white children in the adolescent-onset group only. White children showed a lower intercept with a greater linear rate of change in comparison to the Asian and black children, and a lower quadratic slope compared to the black children only. The model testing differences between mixed ethnicity and white children was nonsymmetric or highly singular. In the next step, group-based trajectory analysis was run separately for the Asian, black, mixed ethnicity, and white children.

**Fig. 1:**
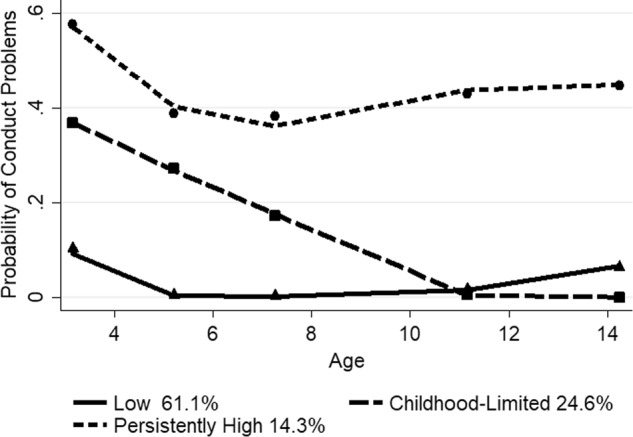
Trajectory groups of conduct problems for Asian children from ages 3 to 14 years Shown are estimated trajectories (lines), observed group means at each age (markers) and estimated group percentages

For Asian children, the three-group, cubic model fit the data best (*N* = 5973). The three-group, quadratic model and four and five-group models showed high estimates and/or were non-symmetric. The mean posterior probability scores ranged from 0.70 to 0.83 for the three-group, quadratic model, with a mean of 0.75, indicating that most fit their assigned trajectory well. Figure 1 depicts the probability of clinically relevant conduct problems for the three trajectory groups of Asian children from ages 3 to 14 years, along with the estimated proportion in each group. The predicted and observed means were similar, indicating a good fit of the model. The low problem group (61.1%) displayed a slightly raised probability of 0.10 at age 3 which then was near zero from age 5, with a slight increase to 0.10 at age 14. There was a childhood-limited group (24.6%), which followed a probability of close to 0.40 at age 3 years, which steadily decreased to near zero by age 11. In a persistently high group, 14.3% showed a relatively high probability of close to 0.60 at age 3, which then dropped to around 0.40 from ages 5 to 14 years.

**Fig. 2:**
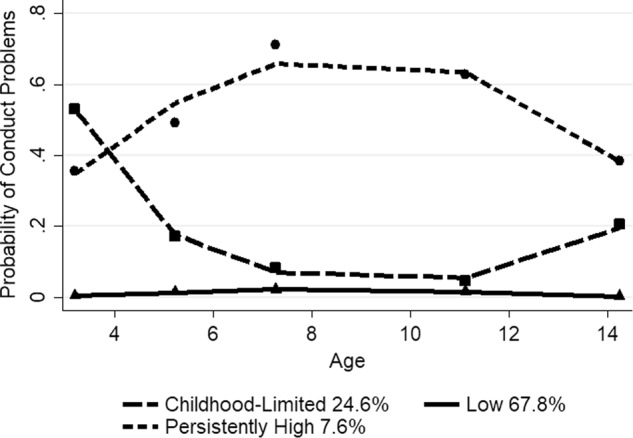
Trajectory groups of conduct problems for black children from ages 3 to 14 years Shown are estimated trajectories (lines), observed group means at each age (markers) and estimated group percentages

For black children, the final model meeting the selection criteria included three-group, quadratic trajectories (*N* = 2033). The BIC score for the three-group, quadratic model (−636.82) was better than for the three-group, cubic model (−647.37). The four and five-group models showed high estimates and/or were non-symmetric. The mean posterior probability scores ranged from 0.73 to 0.83 for the three-group trajectory model, with a mean of 0.79, indicating that most children fit their assigned trajectory well. Figure 2 depicts the probability of clinically relevant conduct problems for the three trajectory groups of black children from ages 3 to 14, along with the estimated percentage in each group. The predicted and observed means were similar, indicating a good fit of the model. The low problem group (67.8%) displayed a near zero probability of conduct problems from ages 3 to 14. There was a childhood-limited group (24.6%), which followed a probability of close to 0.60 at age 3 years, declining to around 0.10 from ages 7 to 11 years, with a slight increase to 0.20 at age 14. A persistently high group (7.6%) showed an increase from 0.40 at age 3 to above 0.60 from ages 7 to 11 years, with a decline to about 0.40 at age 14.

**Fig. 3:**
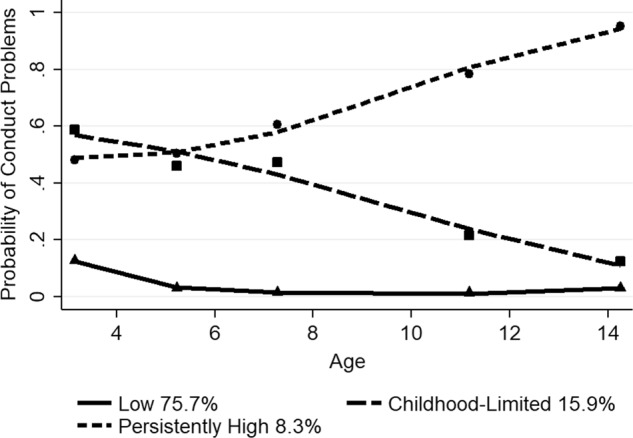
Trajectory groups of conduct problems for mixed ethnicity children from ages 3 to 14 years Shown are estimated trajectories (lines), observed group means at each age (markers) and estimated group percentages

For the mixed ethnicity children, the final model meeting the selection criteria included three-group, quadratic trajectories (*n* = 1905). The BIC score for the three-group, quadratic model (−735.60) was better than the four-group, quadratic model (−748.01). The cubic and five-group models showed a false or singular convergence. The mean posterior probability scores ranged from 0.75 to 0.90 for the three-group, quadratic trajectory model, with a mean of 0.80, indicating that most children fit their assigned trajectory well. Figure 3 depicts the probability of clinically relevant conduct problems for the three trajectory groups of mixed ethnicity children from ages 3 to 14, along with the estimated percentage in each group. The predicted and observed means were similar, indicating a good fit of the model. The low problem group (75.7%) had close to 0.20 probability at age 3, which then fell to a near zero probability of conduct problems from ages 5 to 14. There was a childhood-limited group (15.9%), which showed a probability of close to 0.60 at age 3 years, followed by a steady decline falling to below 0.20 at age 14. A persistently high group (8.3%) showed a moderate probability of about 0.50 from ages 3 to 5 years, then increased steadily to almost 1.0 by age 14.

**Fig. 4:**
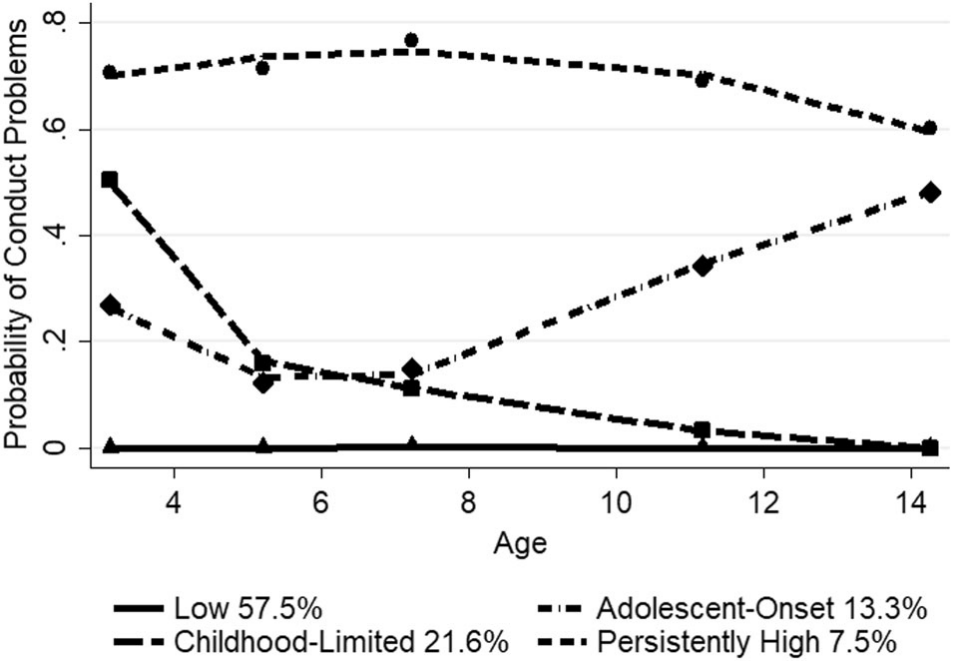
Trajectory groups of conduct problems for white children from ages 3 to 14 years Shown are estimated trajectories (lines), observed group means at each age (markers) and estimated group percentages

For white children, the final model meeting the selection criteria included four-group, cubic trajectories (*n* = 56511). The BIC score for the four-group, cubic model (−18213.21) was better than the four-group, quadratic model (−18218.84). The three and five-group models showed high estimates and/or were non-symmetric. The mean posterior probability scores ranged from 0.74 to 0.81 for the four-group trajectory model, with a mean of 0.78, indicating that most children fit their assigned trajectory well. Figure 4 depicts the probability of clinically relevant conduct problems for the four trajectory groups of white children from ages 3 to 14, along with the estimated percentage in each group. The predicted and observed values were similar, indicating a good fit of the model. The low problem group (57.5%) showed an almost zero probability from ages 3 to 14 years. There was a childhood-limited group (21.6%), which displayed a high probability at age 3 years (close to 0.50) that declined sharply at age 5, steadily falling from age 7 and reaching a zero probability by age 14. There also was an adolescent-onset group (13.3%) showing a moderate probability of 0.30 at age 3, decreasing to below 0.20 from ages 5 to 7 then gradually increasing to near 0.50 by age 14. The persistently high group (7.5%) displayed a high probability from ages 3 to 11 years (around 0.70), with a slight dip to 0.60 at age 14.

### Early Predictors of Conduct Problem Trajectories

Tables 2–5 present the proportions and SD of gender and the early predictors by trajectory group for the Asian, black, mixed ethnicity, and white children, respectively. Superscripts reflect post-hoc analyses, where all possible pairwise differences among the groups were compared. For the Asian children (see Table 2), the persistently high group were more likely to live in households with lower levels of education and income compared to children on the low pathway. Mothers of children on the persistently high and childhood-limited pathways reported more depressive symptoms and less positive child-parent relationships than mothers of children following the low trajectory. Several factors, including gender, social housing, single-parent family, and teenage mothers, did not differentiate the pathways among the Asian children.

For the black children (see Table 3), those following the persistently high and childhood-limited pathways were more likely to be male, have mothers with more depressive symptoms, and experience less positive child-parent relationships compared to children on the low pathway. Highest household education, family income, social housing, single-headed family, and teenage mothers did not differentiate the pathways among the black children.

For the mixed ethnicity children (see Table 4), those following the persistently high pathway experienced more social disadvantage, in terms of lower household education and family income and higher levels of living in social housing, than children on the low pathway. Children on the childhood-limited and persistently high trajectories were more likely to live in single parent families and experience less positive child-parent relationships than those in the low group. The childhood-limited pathway was distinguished from both the low and persistently high groups as having the highest percentage of boys. Teenage mothers and maternal depressive symptoms did not differentiate the pathways among the black children.

For the white children (see Table 5), all of the early markers differentiated the high problem groups from the low group. White children following the childhood-limited and adolescent-onset groups had intermediate levels of most early markers between the low and persistently high groups except for the child-parent relationship, with mothers of children on the childhood-limited pathway reporting a less positive child-parent relationship than mothers of children on the adolescent-onset pathway. White children on the persistently high pathway had the highest levels of risk on all of the early predictors compared to the other groups.

## Discussion

A substantial body of evidence has examined developmental pathways into and out of conduct problems. However, there is a dearth of research assessing whether the same conduct problem pathways are evident in minority ethnic, as in white, populations. Drawing on the UK Millennium Cohort Study, a nationally representative longitudinal study of children born between 2000 and 2002, this study examines differences in group-based trajectories of conduct problems according to broad categories of ethnicity. Using pathways identified in a prior study, including persistently high (8%), childhood-limited (23%), adolescent-onset (13%), and low (56%), significant differences between Asian and white children and black and white children were found. As a result, trajectories of conduct problems were identified separately for Asian, black, mixed ethnicity, and white children. For Asian, black, and mixed ethnicity children, three trajectories were identified: persistently high, childhood-limited, and low, but not adolescent-onset. Although these pathways have similar labels, their patterns and shapes seem to differ among the three ethnic groups. For white children, the same four trajectory groups were identified as in the prior study. Significant risk factors also differed among the groups according to ethnicity, although a less positive child-parent relationship was a significant predictor of the higher problem trajectories for all ethnic groups. Overall, the findings suggest that black and minority ethnic children may follow different developmental pathways of conduct problems than white children, particularly during adolescence, having implications for service use and early intervention.

### Conduct Problem Trajectories

A similar proportion of Asian, black, and white children followed the childhood-limited pathway (around 25%). This proportion is higher than the mixed ethnicity group at 15.9%, which coincides with other population studies identifying 12% of Belgian adolescents (Sentse et al. 2017) and 15% of UK adolescents (Barker and Maughan 2009) as “childhood-limited”. The shape of this pathway somewhat differs among the ethnic groups, with the black and white children reaching below 0.20 by age 5 while the Asian and mixed ethnicity children reach at or below 0.20 by ages 7 and 11, respectively. What is concerning is the slight rise in conduct problems shown for the black children in this group at age 14, suggesting a possible increase in middle to late adolescence.

For all three ethnic groups, there is a stable pathway of children who show persistently high levels of conduct problems from ages 3 to 14 years. Overall, the prevalence rates for black, mixed ethnicity, and white children are similar to prior evidence from international community samples, which show 10% or less of the sample in the persistently high group (Broidy et al. 2003; Côté et al. 2006; Van Lier and Crijnen 2005). However, the prevalence for the Asian children at 14.3% is somewhat higher. There are also differences in the probability of high-risk conduct problems among ethnic groups: the probability is highest for Asian children at age 3 and black children from ages 7 to 11, but remains fairly stable for white children from ages 3 to 14. Most concerning is the steady increase shown for the mixed ethnicity children, reaching near 1.0 by age 14. This finding supports previous research showing an increased risk of conduct problems for mixed ethnicity children in adolescence (Zilanawala et al. 2018). It is possible that this persistently high but increasing pathway for the mixed ethnicity children includes a small percentage with adolescent-onset, who are pushing this trajectory upwards in adolescence. On the other hand, mixed ethnicity children in this group may show heightened vulnerability in early childhood, cascading into escalating problems in adolescence. In either case, this group of mixed ethnicity children is at high-risk of severe conduct problems by the time they reach early to middle adolescence.

For the black and minority ethnicity children, what is missing is the presence of an adolescent-onset group. The adolescent-onset group did not emerge for either the Asian, black, or mixed ethnicity children, indicating that there is not a significant proportion showing low levels in early childhood with onset of high conduct problems in early to middle adolescence. In support of previous studies (Barker and Maughan 2009; Sentse et al. 2017), this study identified the adolescent-onset pathway for white children, with 13.3% of the sample beginning to display high levels of conduct problems from early to middle adolescence. Taking into consideration both the adolescent-onset and persistently high groups, more than 20% of the white sample have at least a 0.50 probability of severe conduct problems at age 14; a percentage that is higher than the black and minority ethnic groups. This coincides with previous findings that black and minority ethnic adolescents in the UK tend to have lower scores on SDQ total difficulties, compared to white adolescents (Fagg et al. 2006, Goodman et al. 2010). The lower prevalence of severe conduct problems for black and minority ethnic adolescents may reflect different cultural values and supportive social networks, although further research is needed to understand the specific processes.

### Early Predictors of Conduct Problem Trajectories

These findings confirm other studies showing that being male, experiencing social disadvantage, having a teenage mother, living in a single parent household, having a mother with depressive symptoms, and having a poor child-parent relationship are significant risk factors of high conduct problem trajectories for white children (Barker and Maughan 2009; Gutman et al. 2019). For both the white and mixed ethnicity children, the persistently high group showed the greatest social disadvantage. White children following this pathway were also distinguished as experiencing the most risky early predictors compared to the other groups; this was not the case for the mixed ethnicity children, who showed no significant differences in having teenage mothers and maternal depressive symptoms. For both the Asian and black children, having a teenage mother, living in social housing, and having a single parent did not distinguish between the high and low problem groups. Interestingly, this does not seem to be related to the prevalence of those risks, as black children were more likely to live in social housing and with a single parent than white children, while Asian children had similar rates of living in social housing but lower rates of living with a single parent compared to white children. The potential negative effects of these early markers for black and minority ethnic children may be reduced by factors related to family life and social support networks, which may buffer their adverse effects on health outcomes (e.g., Dogra et al. 2013). What is also noteworthy is the overrepresentation of males in the higher problem groups for the black children and in the childhood-limited group for mixed ethnicity children, which is not present for the Asian children and seems higher in comparison to the white children. For all ethnic groups, children in the higher problem trajectories had mothers who reported a worse child-parent relationship than those in the low group, highlighting the importance of early parenting intervention.

### Limitations

A number of limitations should be considered. First, this study is exploratory and further studies should be conducted to verify its findings, especially with samples from other countries, using different measures of conduct problems, and for a longer time frame. Second, ethnicity, conduct problems, and risk factors were assessed using parent-reports. This raises the problem of informant and methodical biases and caution is needed when inferring the meaning and generalizability of the results. Another potential issue is that children’s ethnic identity may differ from parents’ reports. Second, respondents were lost between the first and second surveys, which were overrepresented by black and minority ethnic families. Because the predictors of attrition are also predictors of child conduct problems, this sample is likely to under-represent children with the most severe difficulties, as well as family histories with the greatest increase in risk factors. Moreover, it is possible that these predictors interacted with ethnicity status, which also was related to attrition, creating a more complicated pattern of differential attrition. Third, only early markers were assessed in this study; therefore, other potentially important factors for children and adolescents such as the effects of racism were not examined (Astell-Burt et al. 2012; Wallace et al. 2016). Fourth, this study examined data only up to age 14 years. Therefore, ethnic differences may be due to age-related factors and the black and minority ethnic sample may show four trajectories, if followed through adolescence. A final limitation is that this study examines black and minority ethnic children in comparison to white children. This presents the latter group as the invariant, normative benchmark, masking important differences in this group such as geographic region (Goodman et al. 2008). Furthermore, the sample size of the black and minority ethnic groups limits the conclusions that can be drawn from these analyses as a result of reduced statistical power in comparison to the white group. Due to issues with a smaller sample size, this study focuses on broad ethnic groups based on the UK 2011 Census, which ignores differences amongst them. These groups have distinct cultural and religious traditions as well as different migration patterns to the UK, with the primary wave of migration of Black Caribbeans and Indians to the UK occurring in the 1950s and 1960s, for Pakistanis the 1960s and 1970s, Bangladeshis the 1980s, and Black Africans the 1990s. Additional research is needed to understand the broader social and economic factors that may shape these trajectories within and between different ethnic groups and further assess inter-ethnic similarities and differences in their etiology, developmental course, and later outcomes.

## Conclusions

Numerous studies have shown that children tend to follow one of four developmental trajectories of conduct problems: persistently high, childhood-limited, adolescent-onset, and low pathways. Nevertheless, there is a dearth of research examining whether black and minority ethnic children show these same four heterogenous pathways, with similar starting points and rates of change across development. With a nationally representative sample of children born in the new millennium, this study fills this gap, showing that there does not seem to be a significant group of black and minority ethnic children that follow an adolescent-onset pathway. Thus, the developmental taxonomy may not apply to black and minority ethnic children in the UK; they are not characterised with a rebellious group of teenagers who engage in conduct problems beginning in adolescence. Rather, a significant minority have moderate to high levels of conduct problems beginning in early childhood and continuing into adolescence. Particularly concerning is the small group of mixed ethnicity children who show persistently high and increasing levels of conduct problems from early childhood into adolescence. Overall, this study represents a step towards understanding ethnic differences in the developmental pathways of conduct problems, but further research exploring a broader range of health and wellbeing contexts is needed. The importance of addressing this issue reaches across many different levels, not only to acknowledge that distinct groups may experience diverse pathways of development both within and across health and wellbeing, but also for the purposes of reducing existing inequalities in the diagnosis and treatment of mental health problems as well as designing and targeting early intervention for black and minority ethnic children.

## References

[CR1] Alink LR, Egeland B (2013). The roles of antisocial history and emerging adulthood developmental adaptation in predicting adult antisocial behavior. Aggressive Behavior.

[CR2] American Psychiatric Association. (2013). Diagnostic and statistical manual of mental disorders (DSM). 5th edn. Washington, DC: American Psychiatric Association.

[CR3] Astell-Burt T, Maynard MJ, Lenguerrand E, Harding S (2012). Racism, ethnic density and psychological well-being through adolescence: evidence from the determinants of adolescent social well-being and health longitudinal study. Ethnicity & Health.

[CR4] Barker ED, Maughan B (2009). Differentiating early-onset persistent childhood-limited conduct problem youth. American Journal of Psychiatry.

[CR5] Bevilacqua L, Hale D, Barker ED, Viner R (2017). Conduct problems trajectories and psychosocial outcomes: a systematic review and meta-analysis. European Child & Adolescent Psychiatry.

[CR6] Broidy LM, Nagin DS, Tremblay RE, Bates JE, Brame B, Dodge KA, Lynam DR (2003). Developmental trajectories of childhood disruptive behaviors and adolescent delinquency: a six-site, cross-national study. Developmental Psychology.

[CR7] Cabinet Office (2018). UK population by ethnicity. https://www.ethnicity-facts-figures.service.gov.uk/uk-population-by-ethnicity

[CR8] Cavendish W, Nielsen AL, Montague M (2012). Parent attachment, school commitment, and problem behavior trajectories of diverse adolescents. Journal of Adolescence.

[CR9] Côté SM, Vaillancourt T, LeBlanc JC, Nagin DS, Tremblay RE (2006). The development of physical aggression from toddlerhood to pre-adolescence: a nation-wide longitudinal study of Canadian children. Journal of Abnormal Child Psychology.

[CR58] Croft S, Stride C, Maughan B, Rowe R (2015). Validity of the Strengths and Difficulties Questionnaire in Preschool-Aged Children. PEDIATRICS.

[CR10] Department for Work and Pensions (22 March 2018). https://assets.publishing.service.gov.uk/government/uploads/system/uploads/attachment_data/file/691848/income-dynamics-income-movements-and-persistence-of-low-incomes-2015-16.pdf

[CR11] Dogra N, Svirydzenka N, Dugard P, Singh SP, Vostanis P (2013). Characteristics and rates of mental health problems among Indian and White adolescents in two English cities. The British Journal of Psychiatry.

[CR12] Fagg J, Curtis S, Stansfeld S, Congdon P (2006). Psychological distress among adolescents, and its relationship to individual, family and area characteristics in East London. Social science & medicine.

[CR13] Frick PJ (2012). Developmental pathways to conduct disorder: implications for future directions in research, assessment, and treatment. Journal of Clinical Child and Adolescent Psychology.

[CR15] Frick PJ, Viding E (2009). Antisocial behavior from a developmental psychopathology perspective. Development and Psychopathology.

[CR16] Goodman R (1997). The strengths and difficulties questionnaire (SDQ). Journal of Child Psychology and Psychiatry.

[CR17] Goodman R (2001). Psychometric properties of the strengths and difficulties questionnaire. Journal of the American Academy of Child & Adolescent Psychiatry.

[CR18] Goodman R, Ford T, Simmons H, Gatward R, Meltzer H (2000). Using the strengths and difficulties questionnaire (SDQ) to screen for child psychiatric disorders in a community sample. British Journal of Psychiatry.

[CR19] Goodman A, Patel V, Leon DA (2010). Why do British Indian children have an apparent mental health advantage?. Journal of Child Psychology and Psychiatry.

[CR20] Goodman A, Patel V, Leon DA (2008). Child mental health differences amongst ethnic groups in Britain: a systematic review. BMC Public Health.

[CR21] Green H, McGinnity Á, Meltzer H, Ford T, Goodman R (2005). Mental health of children and young people in Great Britain, 2004.

[CR22] Gutman LM, Joshi H, Khan L, Schoon I (2018). Children of the millennium..

[CR23] Gutman LM, Joshi H, Schoon I (2019). Developmental trajectories of conduct problems and cumulative risk from early childhood to adolescence. Journal of Youth and Adolescence.

[CR24] Gutman LM, Joshi H, Parsonage M, Schoon I (2018). Gender-specific trajectories of conduct problems from ages 3 to 11. Journal of Abnormal Child Psychology.

[CR25] Gutman L, Joshi H, Parsonage M, Schoon I (2015). Children of the new century. Mental health findings from the Millennium Cohort Study..

[CR26] Hansen, K. (2014). Millennium cohort study: a guide to the data sets. London: UCL Institute of Education.

[CR29] Jones BL, Nagin DS (2013). A note on a stata plugin for estimating group-based trajectory models. Sociological Methods & Research.

[CR30] Joshi H, Fitzsimons E (2016). The millennium cohort study: the making of a multi-purpose resource for social science and policy. Longitudinal and Life Course Studies.

[CR60] Kessler RC, Andrews G, Colpe LJ, Hiripi E, Mroczek DK, Normand S-LT, Walters EE, Zaslavsky AM (2002). Short screening scales to monitor population prevalences and trends in non-specific psychological distress. Psychological Medicine.

[CR57] Ketende, S. C., & Joshi, H. (2008). Technical appendix to Chapter 12 Income and poverty, In K. Hansen, & H. Joshi (eds), *Millennium Cohort Study, Third Survey: Ausers Guide to Initial Findings*. Centre for Longitudinal Studies, Institute of Education, London.

[CR31] Kretschmer T, Hickman M, Doerner R, Emond A, Lewis G, Macleod J, Heron J (2014). Outcomes of childhood conduct problem trajectories in early adulthood: findings from the ALSPAC study. European Child & Adolescent Psychiatry.

[CR32] Klineberg E, Clark C, Bhui KS, Haines MM, Viner RM, Head J, Stansfeld SA (2006). Social support, ethnicity and mental health in adolescents. Social Psychiatry and Psychiatric Epidemiology.

[CR33] Mak W, Rosenblatt A (2002). Demographic influences on psychiatric diagnoses among youth served in California systems of care. Journal of Child and Family Studies.

[CR34] Maynard MJ, Harding S (2010). Perceived parenting and psychological well‐being in UK ethnic minority adolescents. Child: Care, Health and Development.

[CR35] Maynard MJ, Harding S, Minnis H (2007). Psychological well-being in Black Caribbean, Black African, and white adolescents in the UK Medical Research Council DASH study. Social pSychiatry And Psychiatric Epidemiology.

[CR36] Miner JL, Clarke-Stewart KA (2008). Trajectories of externalizing behavior from age 2 to age 9: relations with gender, temperament, ethnicity, parenting, and rater. Developmental Psychology.

[CR37] Moffitt TE (1993). Adolescence-limited and life-course persistint antisocial behavior. A developmental taxonomy. Psychological Review.

[CR38] Moffitt, T. E. (2006). Life-course-persistent versus adolescence-limited antisocial behavior. In D. Cicchetti & D.J. Cohen (Eds.). Developmental psychopathology: risk, disorder, and adaptation vol. 3, 2nd ed., (pp. 570–598). Hoboken, NJ: John Wiley & Sons Inc.

[CR56] Meltzer H, Gatward R, Goodman R, Ford T (2009). Mental health of children and adolescents in Great Britain. International Review of Psychiatry.

[CR39] Nagin D (2005). Group-based modeling of development..

[CR40] Nagin DS, Odgers C (2010). Group-based trajectory modeling in clinical research. Annual Review Clinical Psychology.

[CR41] National Health Service (24 January 2018). https://www.ethnicity-facts-figures.service.gov.uk/health/physical-and-mental-health/adults-experiencing-common-mental-disorders/latest.

[CR42] Nguyen L, Huang LN, Arganza GF, Liao Q (2007). The influence of race and ethnicity on psychiatric diagnoses and clinical characteristics of children and adolescents in children’s services. Cultural Diversity and Ethnic Minority Psychology.

[CR55] Odgers, C. L., Moffitt, T. E., Broadbent, J. M., Dickson, N., Hancox, R. J., Harrington, H., Poulton, R., Sears, M. R. Thomson, W. M., & Caspi, A. (2008). Female and male antisocial trajectories: From childhood origins to adult outcomes. *Development and Psychopathology**20*(2):673–716. 10.1017/S0954579408000333 18423100

[CR43] Office for National Statistics (2009). Final recommended questions for the 2011 Census in England and Wales: Ethnic Group. https://cy.ons.gov.uk/census/2011census/howourcensusworks/howweplannedthe2011census/questionnairedevelopment/finalisingthe2011questionnaire.

[CR62] Pianta, R. C. (1995). Child-Parent Relationship Scale, Charlottesville. VA: University of Virginia.

[CR59] Schafer JL, Graham JW (2002). Missing data: Our view of the state of the art. Psychological Methods.

[CR46] Sentse M, Kretschmer T, de Haan A, Prinzie P (2017). Conduct problem trajectories between age 4 and 17 and their association with behavioral adjustment in emerging adulthood. Journal of Youth and Adolescence.

[CR47] Shaw DS (2013). Future directions for research on the development and prevention of early conduct problems. Journal of Clinical Child & Adolescent Psychology.

[CR48] Shaw DS, Hyde LW, Brennan LM (2012). Early predictors of boys’ antisocial trajectories. Development and psychopathology.

[CR49] Van Lier PA, Crijnen AA (2005). Trajectories of peer-nominated aggression: risk status, predictors and outcomes. Journal of Abnormal Child Psychology.

[CR50] Wallace S, Nazroo J, Bécares L (2016). Cumulative effect of racial discrimination on the mental health of ethnic minorities in the United Kingdom. American Journal of Public Health.

[CR51] Wickham S, Whitehead M, Taylor-Robinson D, Barr B (2017). The effect of a transition into poverty on child and maternal mental health: a longitudinal analysis of the UK Millennium Cohort Study. The Lancet Public Health.

[CR52] Xie H, Drabick DA, Chen D (2011). Developmental trajectories of aggression from late childhood through adolescence: similarities and differences across gender. Aggressive Behavior.

[CR54] Zilanawala A, Sacker A, Kelly Y (2018). Mixed ethnicity and behavioural problems in the Millennium Cohort Study. Archives of Disease in Childhood.

